# Methyl 3-amino­but-2-enoate

**DOI:** 10.1107/S160053681202288X

**Published:** 2012-05-31

**Authors:** Xiao Wang, Li-Zhu Zhang

**Affiliations:** a Department of Applied Chemistry, Harbin Institute of Technology, Harbin, Heilongjiang 150001, People’s Republic of China

## Abstract

The title compound, C_5_H_9_NO_2_, is almost planar (r.m.s. deviation for the non-H atoms = 0.036 Å) and an intra­molecular N—H⋯O hydrogen bond generates an *S*(6) ring. In the crystal, N—H⋯O inter­actions link the mol­ecules into *C*(6) chains propagating along [010].

## Related literature

For further synthetic details, see: Rakshit *et al.* (2010 ▶); Vanden Eynde *et al.* (1995 ▶).
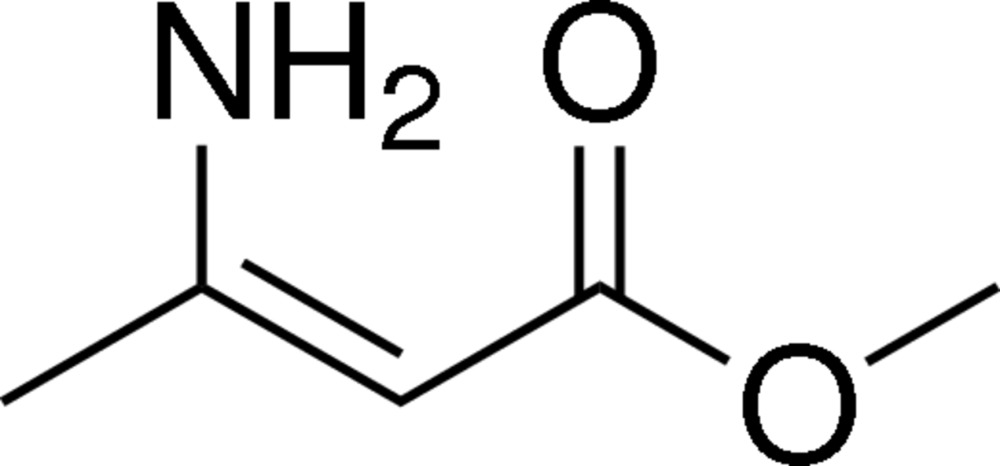


## Experimental

### 

#### Crystal data

C_5_H_9_NO_2_
*M* *_r_* = 115.13
Monoclinic, 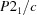

*a* = 8.3020 (12) Å
*b* = 9.7232 (14) Å
*c* = 7.665 (1) Å
β = 97.855 (13)°
*V* = 612.93 (15) Å^3^
*Z* = 4
Cu *K*α radiation
μ = 0.81 mm^−1^
*T* = 113 K
0.18 × 0.16 × 0.10 mm

#### Data collection

Rigaku Saturn944 CCD diffractometer
Absorption correction: multi-scan (*CrystalClear*; Rigaku, 2009 ▶) *T*_min_ = 0.868, *T*_max_ = 0.924
6605 measured reflections
1175 independent reflections
1019 reflections with *I* > 2σ(*I*)
*R*_int_ = 0.071

#### Refinement

*R*[*F* ^2^ > 2σ(*F* ^2^)] = 0.067
*wR*(*F* ^2^) = 0.141
*S* = 1.20
1175 reflections
84 parameters
H atoms treated by a mixture of independent and constrained refinement
Δρ_max_ = 0.36 e Å^−3^
Δρ_min_ = −0.51 e Å^−3^

### 

Data collection: *CrystalClear* (Rigaku, 2009 ▶); cell refinement: *CrystalClear*; data reduction: *CrystalClear*; program(s) used to solve structure: *SHELXS97* (Sheldrick, 2008 ▶); program(s) used to refine structure: *SHELXL97* (Sheldrick, 2008 ▶); molecular graphics: *SHELXTL* (Sheldrick, 2008 ▶); software used to prepare material for publication: *SHELXL97*.

## Supplementary Material

Crystal structure: contains datablock(s) global, I. DOI: 10.1107/S160053681202288X/hb6788sup1.cif

Structure factors: contains datablock(s) I. DOI: 10.1107/S160053681202288X/hb6788Isup2.hkl

Supplementary material file. DOI: 10.1107/S160053681202288X/hb6788Isup3.cml

Additional supplementary materials:  crystallographic information; 3D view; checkCIF report

## Figures and Tables

**Table 1:** Hydrogen-bond geometry (Å, °)

*D*—H⋯*A*	*D*—H	H⋯*A*	*D*⋯*A*	*D*—H⋯*A*
N1—H1*A*⋯O1^i^	0.84 (2)	2.05 (2)	2.8778 (16)	168.9 (19)
N1—H1*B*⋯O1	0.89 (2)	2.08 (2)	2.7168 (16)	127.7 (15)

Symmetry code: (i) 
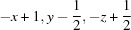
.

**Fig. 1.**
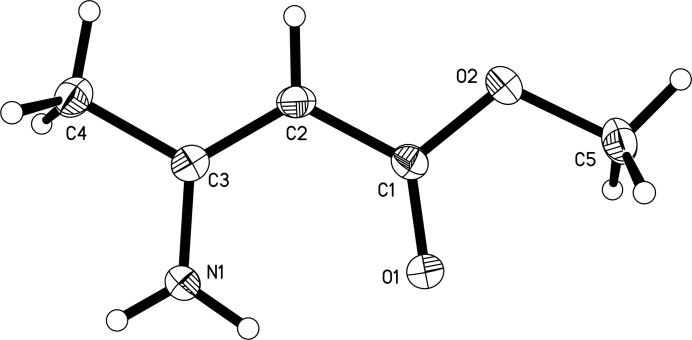
The molecular structure of (I), with 30% probability displacement ellipsoids.

**Fig. 2.**
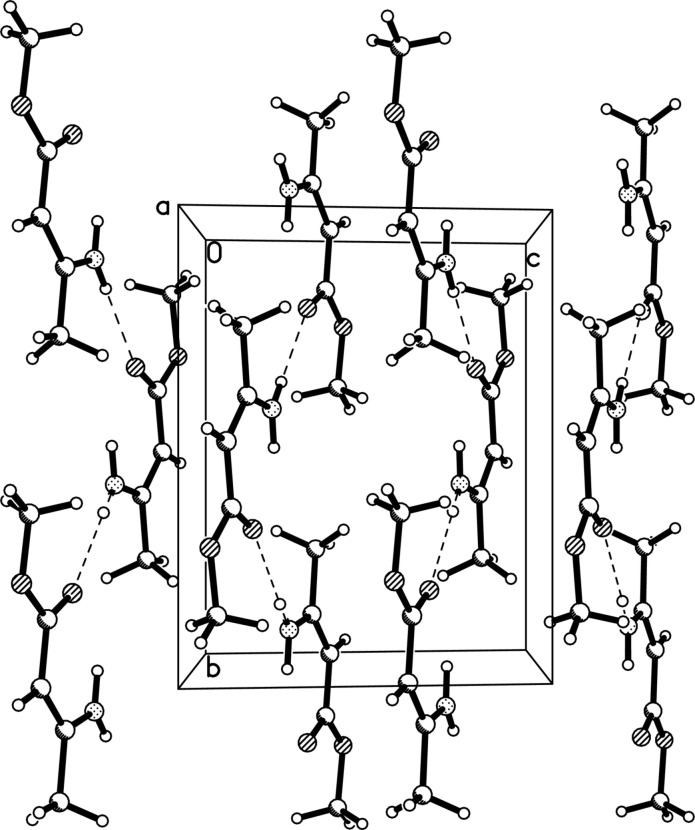
Packing diagram for (I).

## supplementary crystallographic information

### Comment

Nifedipine was found to be a highly effective calcium antagonist. Consequently,many compounds which were similar in structure to nifedipine have already been used as therapeutic agents for treatment of cerebral circulatory disorder,hypertension and so on. The title compound (I) is an intermediate for the synthesis of this family of compounds and its structure is reported here. As shown in Fig. 1, in each molecular unit, almost non-hydrogen atoms in the same plane, and the deviation is 0.036 nm. The length of the double bond is slightly longer than the normal double bond of ethylene likewise, the bond between carbon and nitrogen are shorter than normal C—N bond. A short intermolecular N—H···O interaction (Table 1) occurs [symmetry code:(i)-*x* + 1,*y* - 1/2,-*z* + 1/2], and relatively strong intramolecular N—H···O hydrogen bonds also exists.

### Experimental

Impoved from the published methods by Rakshit *et al.* (2010) and Vanden Eynde *et al.* (1995) a modification of the synthetic procedure was used to prepare the title compound from methyl acetoacetate and ammonium acetate. Colorless prisms of (I) were obtained by recrystallizing from a ethyl acetate solution. mp: 355 K. Analysis, calculated for C_5_H_9_NO_2_: C 52.16, H 7.88, N 12.17; found: C 52.15, H 7.87, N 12.16.

### Crystal data

**Table:**

C_5_H_9_NO_2_	*D*_x_ = 1.248 Mg m^−^^3^
*M**_r_* = 115.13	Melting point: 355 K
Monoclinic, *P*2_1_/*c*	Cu *K*α radiation, λ = 1.54187 Å
*a* = 8.3020 (12) Å	Cell parameters from 670 reflections
*b* = 9.7232 (14) Å	θ = 27.9–71.6°
*c* = 7.665 (1) Å	µ = 0.81 mm^−^^1^
β = 97.855 (13)°	*T* = 113 K
*V* = 612.93 (15) Å^3^	Prism, colorless
*Z* = 4	0.18 × 0.16 × 0.10 mm
*F*(000) = 248	

### Data collection

**Table:**

Rigaku Saturn944 CCD diffractometer	1175 independent reflections
Radiation source: fine-focus sealed tube	1019 reflections with *I* > 2σ(*I*)
Multilayer monochromator	*R*_int_ = 0.071
Detector resolution: 14.629 pixels mm^-1^	θ_max_ = 71.9°, θ_min_ = 5.4°
ω scans	*h* = −10→10
Absorption correction: multi-scan (*CrystalClear*; Rigaku, 2009)	*k* = −11→9
*T*_min_ = 0.868, *T*_max_ = 0.924	*l* = −9→9
6605 measured reflections	

### Refinement

**Table:**

Refinement on *F*^2^	Secondary atom site location: difference Fourier map
Least-squares matrix: full	Hydrogen site location: inferred from neighbouring sites
*R*[*F*^2^ > 2σ(*F*^2^)] = 0.067	H atoms treated by a mixture of independent and constrained refinement
*wR*(*F*^2^) = 0.141	*w* = 1/[σ^2^(*F*_o_^2^) + (0.0954*P*)^2^] where *P* = (*F*_o_^2^ + 2*F*_c_^2^)/3
*S* = 1.20	(Δ/σ)_max_ < 0.001
1175 reflections	Δρ_max_ = 0.36 e Å^−^^3^
84 parameters	Δρ_min_ = −0.51 e Å^−^^3^
0 restraints	Extinction correction: *SHELXL97* (Sheldrick, 2008), Fc^*^=kFc[1+0.001xFc^2^λ^3^/sin(2θ)]^-1/4^
Primary atom site location: structure-invariant direct methods	Extinction coefficient: 0.32 (2)

### Special details

**Table:**

Geometry. All e.s.d.'s (except the e.s.d. in the dihedral angle between two l.s. planes) are estimated using the full covariance matrix. The cell e.s.d.'s are taken into account individually in the estimation of e.s.d.'s in distances, angles and torsion angles; correlations between e.s.d.'s in cell parameters are only used when they are defined by crystal symmetry. An approximate (isotropic) treatment of cell e.s.d.'s is used for estimating e.s.d.'s involving l.s. planes.
Refinement. Refinement of *F*^2^ against ALL reflections. The weighted *R*-factor *wR* and goodness of fit *S* are based on *F*^2^, conventional *R*-factors *R* are based on *F*, with *F* set to zero for negative *F*^2^. The threshold expression of *F*^2^ > σ(*F*^2^) is used only for calculating *R*-factors(gt) *etc*. and is not relevant to the choice of reflections for refinement. *R*-factors based on *F*^2^ are statistically about twice as large as those based on *F*, and *R*- factors based on ALL data will be even larger.

### Fractional atomic coordinates and isotropic or equivalent isotropic displacement parameters (Å^2^)

**Table:**

	*x*	*y*	*z*	*U*_iso_*/*U*_eq_	
O1	0.36825 (11)	0.18110 (10)	0.32340 (12)	0.0327 (4)	
O2	0.12958 (12)	0.21372 (10)	0.42596 (13)	0.0357 (4)	
N1	0.45092 (13)	−0.08490 (14)	0.27315 (14)	0.0320 (4)	
C1	0.24663 (14)	0.13160 (14)	0.37507 (15)	0.0279 (4)	
C2	0.21064 (14)	−0.01182 (15)	0.38684 (15)	0.0294 (4)	
H2	0.1142	−0.0387	0.4317	0.035*	
C3	0.31094 (14)	−0.11148 (14)	0.33542 (15)	0.0285 (4)	
C4	0.26670 (18)	−0.26132 (15)	0.34523 (18)	0.0348 (4)	
H4A	0.2644	−0.3035	0.2288	0.042*	
H4B	0.1592	−0.2697	0.3836	0.042*	
H4C	0.3477	−0.3083	0.4297	0.042*	
C5	0.15619 (19)	0.35898 (16)	0.4094 (2)	0.0400 (5)	
H5A	0.2563	0.3854	0.4849	0.048*	
H5B	0.0641	0.4096	0.4455	0.048*	
H5C	0.1663	0.3810	0.2866	0.048*	
H1B	0.487 (2)	0.002 (2)	0.269 (3)	0.048 (5)*	
H1A	0.515 (2)	−0.147 (2)	0.249 (2)	0.046 (5)*	

### Atomic displacement parameters (Å^2^)

**Table:**

	*U*^11^	*U*^22^	*U*^33^	*U*^12^	*U*^13^	*U*^23^
O1	0.0298 (6)	0.0295 (6)	0.0403 (6)	−0.0019 (4)	0.0102 (4)	−0.0002 (4)
O2	0.0328 (6)	0.0327 (7)	0.0437 (6)	0.0049 (4)	0.0128 (4)	−0.0031 (4)
N1	0.0287 (6)	0.0280 (8)	0.0409 (7)	0.0012 (5)	0.0109 (5)	−0.0021 (5)
C1	0.0263 (6)	0.0312 (9)	0.0262 (6)	0.0009 (5)	0.0040 (5)	−0.0015 (5)
C2	0.0259 (6)	0.0325 (9)	0.0311 (7)	−0.0039 (5)	0.0080 (5)	−0.0006 (5)
C3	0.0299 (7)	0.0301 (8)	0.0252 (6)	−0.0030 (5)	0.0024 (5)	−0.0002 (5)
C4	0.0423 (8)	0.0294 (8)	0.0335 (7)	−0.0043 (6)	0.0078 (6)	−0.0007 (5)
C5	0.0457 (8)	0.0316 (9)	0.0436 (8)	0.0095 (6)	0.0097 (6)	−0.0031 (6)

### Geometric parameters (Å, º)

**Table:**

O1—C1	1.2317 (16)	C2—H2	0.9500
O2—C1	1.3557 (15)	C3—C4	1.5069 (18)
O2—C5	1.4379 (18)	C4—H4A	0.9800
N1—C3	1.3404 (17)	C4—H4B	0.9800
N1—H1B	0.89 (2)	C4—H4C	0.9800
N1—H1A	0.84 (2)	C5—H5A	0.9800
C1—C2	1.432 (2)	C5—H5B	0.9800
C2—C3	1.3702 (19)	C5—H5C	0.9800
			
C1—O2—C5	115.36 (11)	C3—C4—H4A	109.5
C3—N1—H1B	120.4 (12)	C3—C4—H4B	109.5
C3—N1—H1A	123.3 (14)	H4A—C4—H4B	109.5
H1B—N1—H1A	115.8 (18)	C3—C4—H4C	109.5
O1—C1—O2	120.91 (13)	H4A—C4—H4C	109.5
O1—C1—C2	126.03 (12)	H4B—C4—H4C	109.5
O2—C1—C2	113.05 (11)	O2—C5—H5A	109.5
C3—C2—C1	122.04 (12)	O2—C5—H5B	109.5
C3—C2—H2	119.0	H5A—C5—H5B	109.5
C1—C2—H2	119.0	O2—C5—H5C	109.5
N1—C3—C2	123.82 (13)	H5A—C5—H5C	109.5
N1—C3—C4	115.67 (12)	H5B—C5—H5C	109.5
C2—C3—C4	120.50 (12)		
			
C5—O2—C1—O1	1.92 (16)	O2—C1—C2—C3	177.61 (10)
C5—O2—C1—C2	−177.39 (11)	C1—C2—C3—N1	1.13 (19)
O1—C1—C2—C3	−1.7 (2)	C1—C2—C3—C4	−178.35 (10)

### Hydrogen-bond geometry (Å, º)

**Table:**

*D*—H···*A*	*D*—H	H···*A*	*D*···*A*	*D*—H···*A*
N1—H1*A*···O1^i^	0.84 (2)	2.05 (2)	2.8778 (16)	168.9 (19)
N1—H1*B*···O1	0.89 (2)	2.08 (2)	2.7168 (16)	127.7 (15)

Symmetry code: (i) −*x*+1, *y*−1/2, −*z*+1/2.
